# Evaluation of the effects of blue-enriched white light on cognitive performance, arousal, and overall appreciation of lighting

**DOI:** 10.3389/fpubh.2024.1390614

**Published:** 2024-05-15

**Authors:** Valérie Gagné, Rose Turgeon, Valérie Jomphe, Claude M. H. Demers, Marc Hébert

**Affiliations:** ^1^CERVO Brain Research Centre, Centre Intégré Universitaire de Santé et des Services Sociaux de la Capitale Nationale, Quebec, QC, Canada; ^2^École d’Architecture, Faculté d’aménagement, d’architecture, d’art et de design, Université Laval, Quebec, QC, Canada; ^3^Département d’Ophtalmologie et Otorhinolaryngologie – Chirurgie Cervico-Faciale, Faculté de Médecine, Université Laval, Quebec, QC, Canada

**Keywords:** ambient light, photopic, melanopic, sleepiness, concentration, comfort, visual performance

## Abstract

**Introduction:**

Light’s non-visual effects on the biological clock, cognitive performance, alertness, and mental health are getting more recognized. These are primarily driven by blue light, which triggers specific retinal cells containing melanopsin. Traditionally, research on light has relied on correlated color temperature (CCT) as a metric of its biological influence, given that bluer light corresponds to higher Kelvin values. However, CCT proves to be an inadequate proxy of light’s biological effects. A more precise metric is melanopic Equivalent Daylight Illuminance (mel-EDI), which aligns with melanopsin spectrum. Studies have reported positive cognitive impacts of blue-enriched white light. It’s unclear if the mixed results are due to different mel-EDI levels since this factor wasn’t assessed.

**Method:**

Given recent recommendations from experts to aim for at least 250 mel-EDI exposure daily for cognitive benefits, our aim was to assess if a 50-minute exposure to LED light with 250 mel-EDI could enhance concentration and alertness, without affecting visual performance or comfort compared to conventional lighting producing around 150 mel-EDI. To ensure mel-EDI’s impact, photopic lux levels were kept constant across conditions. Conditions were counterbalanced, parameters included subjective sleepiness (KSS; Karolinska Sleepiness Scale), concentration (d2-R test), visual performance (FrACT; Freiburg Visual Acuity and Contrast Test), general appreciation (VAS; Visual Analogous Scale), preferences and comfort (modified OLS; Office Lighting Survey).

**Results:**

The experimental light significantly reduced sleepiness (*p* = 0.03, Cohen’s d = 0.42) and also decreased contrast sensitivity (*p* = 0.01, Cohen’s d = 0.50). The conventional light was found to be more comfortable (*p* = 0.002, Cohen’s d = 0.62), cheerful (*p* = 0.02, Cohen’s d = 0.46) and pleasant (*p* = 0.005, Cohen’s d = 0.55) while the experimental light was perceived as brighter (*p* = 0.004, Cohen’s d = 0.58) and tended to be more stimulating (*p* = 0.10). Notably, there was a preference for conventional lighting (*p* = 0.004, Cohen’s d=0.56) and concentration was equally improved in both conditions.

**Discussion:**

Despite the lack of further improvement in concentration from exposure to blue-enriched light, given the observed benefits in terms of vigilance, further research over an extended period would be justified. These findings could subsequently motivate cognitive optimization through lighting for workers that would benefit from artificial lighting such as in northern regions.

## 1 Introduction

The significance of light extends beyond vision to influencing our biological clock, cognitive abilities, alertness, sleep, and overall well-being (1–3). These non-visual effects are particularly driven by blue light, stimulating a subset of intrinsically photosensitive retinal ganglion cells (ipRGCs) that contain the photopigment melanopsin (4–7). However, in modern settings where many work indoors under artificial lighting, there’s a risk of blue light deficiency, especially for night shift workers and those in high-latitude regions with limited natural daylight (8–10). The availability of natural daylight significantly diminishes during winter in northern regions like Nunavik, positioned above 55°N latitude, where light availability is just over 5 h compared to 9 h in Montreal (11, 12). Indigenous populations in Arctic areas may adapt better to these seasonal changes through increased outdoor activity, but fly-in fly-out workers, often indoors for extended periods, may face a deficit in blue light exposure. This deficiency can notably impact their biological clock entrainment, cognitive performance and overall well-being (10, 13).

Most studies investigating light’s non-visual effects have relied widely on correlated color temperature (CCT), measured in Kelvin, to gauge its biological impact. CCT essentially reflects the color of light emitted by a heated black body; the higher the CCT, the bluer the light appears (14). However recent findings from Esposito and Houser (15) indicate that CCT is not a reliable measure of light’s biological potency. A more recent advancement is the measurement of melanopic lux, which aligns with the sensitivity spectrum of intrinsically photosensitive retinal ganglion cells (ipRGCs) (16). Unlike traditional photopic lux, which pertains to vision-related photoreceptors, melanopic lux represents a new frontier in evaluating light’s circadian effects. The current recommendation suggests exposure to at least 250 lux of melanopic Equivalent Daylight Illuminance (mel-EDI) during daylight hours (17, 18).

In an effort to better stimulate the melanopsin system, blue-enriched white lights have been developed and tested over the years. Viola et al. (19), compared office workers on one floor exposed to conventional fluorescent lighting (4,000 K) with another floor exposed to blue enriched white light (17,000 K). After one month of exposure, they observed a significant improvement in alertness, mood, performance, and concentration, as well as a decrease in evening sleepiness, irritability, and eye discomfort with the 17,000 K lighting. Keis et al. (20) studied the acute and chronic effects of blue-enriched white light in students’ performance. Results showed that compared to the group exposed to conventional lighting (3,000 K), the group exposed to 5,500 K for 45 min experienced enhance concentration performance, whereas long-term exposure (5 weeks) led to improved cognitive speed performance. A study conducted in Malaysia with 47 medical students yielded intriguing results in terms of subjective preference versus performance and alertness levels. After a 50-min exposure, performance levels and subjective alertness were significantly higher under 6,500 K lighting, followed by 4,000 K lighting, when compared to 3,000 K lighting. Interestingly, despite the superior cognitive performance observed under the cooler lighting temperature, students expressed a preference for the 4,000 K lighting, primarily due to its enhanced visual comfort (21). Choi et al. (22) demonstrated that exposure to 6,500 K LED lighting as opposed to 3,500 K LED lighting, led to a reduction in sleepiness among university students after 60 min of exposure. Similarly, Shamsul et al. (21) also showed in university students exposed to different lighting, provided with a virtual reality headset representing an auditorium, that a 6,500 K environment resulted in superior attention and memory performance after 60 min of exposure, compared to 4,000 K or 3,000 K lighting. Collectively, these studies suggest a consistent alignment in indicating that blue-enriched light can yield to beneficial effects, even under short term exposures. This conclusion is further reinforced by a recent meta-analysis which emphasizes that exposure to high correlated color temperature (kelvin) lighting during the day enhances both subjective and objective arousal (23).

While CCT provides a visual indication of how yellow or blue a light source appears, it does not accurately quantify melanopic lux levels, as discussed earlier. This means that lighting sources with the same CCT values may affect ipRGCs differently. In contrast, melanopic Equivalent Daylight Illuminance (mel-EDI) offers a more precise metric that directly corresponds to the stimulation experienced by ipRGC photoreceptors (15, 24). Despite the widespread use of Kelvin as a proxy for characterizing light, the limitations highlighted here underscore the necessity of adopting mel-EDI, particularly in emerging clinical trials where precise biological impact assessment is crucial (15, 24, 25). Additionally, inconsistencies in maintaining constant photopic lux levels across different lighting conditions further complicate attributing observed effects solely to melanopic lux, considering that photopic lux can also influence cognitive performance (26).

Additionally, while blue-enhanced lighting has shown positive effects, there is a noted preference for conventional fluorescent lamps over the bluish appearance created by these lights, as indicated by Shamsul et al. (21). This decrease in satisfaction could potentially impact the quality of work life for workers and consequently affect their efficiency (27). The emergence of LED (light-emitting diode) lighting technology now allows for achieving a whiter appearance despite higher melanopic levels. LED lighting naturally includes a peak in the blue range (around 460 nm), closely matching the sensitivity peak of melanopsin cells (at 480 nm), while also emitting in the green and red wavelengths, resulting in a more natural-looking light. In fact, a study has demonstrated a short-term improvement in alertness and a preference for an LED-lit environment over a compact fluorescent-lit environment, regardless of cool or warm lighting (28).

We aimed to assess the impact of LED-based lighting with a melanopic EDI of 250 lux compared to lower levels (around 100 lux) on cognitive function during a 50-min exposure. Both lighting setups were achieved using LED fluorescent-like office lighting (5,000 K) and conventional fluorescent lighting (3,500 K), with consistent photopic lux levels. Our goal was to show that the higher melanopic EDI could enhance concentration and arousal without affecting visual comfort, acuity, or contrast perception.

## 2 Materials and methods

### 2.1 Study population

This crossover repeated-measures experimental study design involved 30 healthy adults (19 females, mean age = 23.4 years, range 19–33 years). The sample size was representative of the university population, where recruitment was easier. The young age groups helped to reduce age-related yellowing of the lens of the eye which could increase the variability in terms of retinal light response to enhanced blue white light (29).

The first experimental condition was randomly drawn and though participants were informed on the objectives of the study, they had no intake on which room corresponded to the blue-enriched lighting. Exclusion criteria were: Dyslexia—Presence of a major psychiatric diagnosis (schizophrenia or other psychotic affective disorder, bipolar, depression)—Being diagnosed with retinal disease (uncorrectable 20/20 vision, macular degeneration, cataracts, glaucoma, diabetic retinopathy, etc.), while wearing glasses was not an exclusion factor.—Any disability that may prevent completion of the procedure (brain injury within the last three months or head trauma requiring consultation)—Pregnant women—Night shift workers (unless inactive for the last two months)—Recent travel to a location more than two time zones within the last month—Taking medication (including antihistamines) that may impact alertness levels. Participants who consume caffeine, alcohol, cannabis, and tobacco were not excluded, but were instructed not to use any on the day of the assessments. This approach aimed to isolate the effects of lighting stimulation from the potential influence of other exogenous substances. While regular caffeine drinkers might experience an impact on their performance, replicating this criterion on both examination days enabled us to assess the effects without the confounding influence of other substances. This strategy contributes to a more focused examination of the specific impact of light exposure on cognitive performance and perception. Any other drug use, however, was an exclusion criterion. Each participant signed a consent form approved by the Centre Intégré Universitaire de Santé et des Services Sociaux de la Capitale-Nationale (CIUSSS-CN) Neuroscience and Mental Health Research Ethics Committee.

### 2.2 Study design

The data collection spanned 6 weeks from mid-November to end of December 2021 in Quebec City, with sessions conducted between 8 am and 6 pm through the day. All participants were exposed to the two lighting conditions (experimental, LED blue-enriched white light at 5000 K and control current office fluorescent white light at 3,500 K) in a counterbalanced order. Each exposure was performed on a different day to avoid any carry-over effect but at the same time of day, to counteract any circadian influence on variables such as mood and arousal (30). Participants were asked to have a minimum of 1 day and a maximum of 31 days between the two exhibitions. Light exposure took place in two adjacent rooms, which were freshly painted in white prior to the experiment. Each session began with a 20-min period in which the participant was exposed to very dim (about 25 lux, 3,000 K) non-stimulating lighting while completing descriptive questionnaires. This period was considered a pre-lighting condition, allowing for a reset of the ipRGC stimulation and thus creating a similar baseline for each trial day. The rationale behind the 20-min period is in line with the brain activation theory where 20 min of exposure would be sufficient to activate both cortical and subcortical brain regions (3, 31). We therefore assumed that 20 min in dim light would be sufficient to deactivate the retinal melanopsin cells. Following the pre-condition period, the participants were exposed to one of the two lighting conditions for 50 min.

### 2.3 Room description

The dimensions of the control condition room were 2.83 m × 3.00 m, which was very similar to the experimental room 2.83 m × 2.62 m. None of the rooms had windows. The office furniture (brand new) in each room was the same and positioned in the same way, so that the exposure to light was similar and the walls remained bare, to limit the qualitative influence of a certain type of decor on the overall assessment of the lighting environment evaluated by the questionnaire. Finally, the wall color of each room was white (reflectance about 80%) to limit the absorption of light by a particular color. Even though the arrangement of the desks and the lack of windows does not comply with architectural and ergonomic standards, this arrangement permitted to have a perfect control over the lighting with no interference from natural light.

### 2.4 Lighting

**Figure 1:**
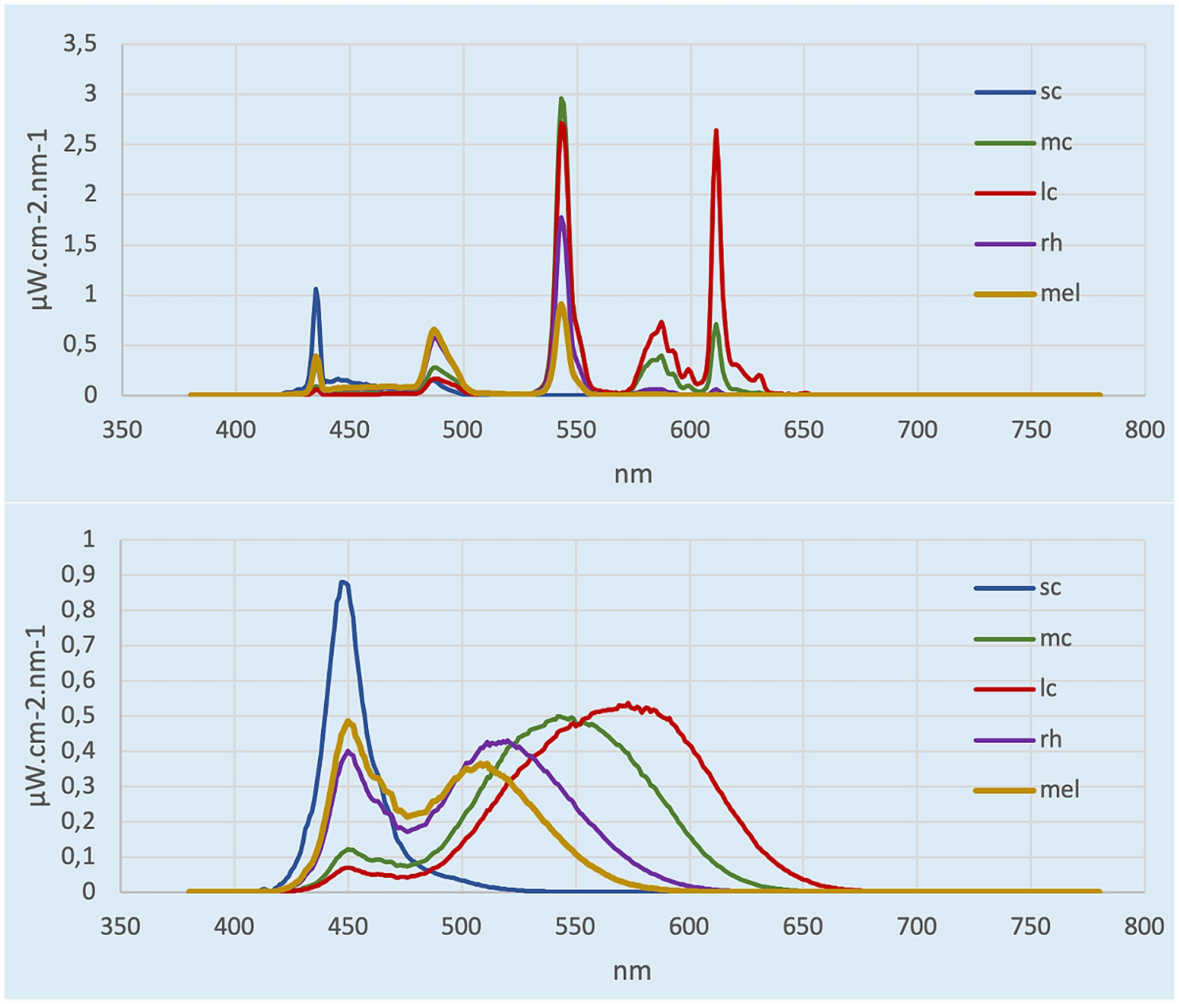
α-opic spectra calculated from CIE toolbox. Top represents the control room (3,500 K, ~150 mel-EDI), bottom represents experimental room (5,000 K, ~260 mel-EDI).

**Figure 2:**
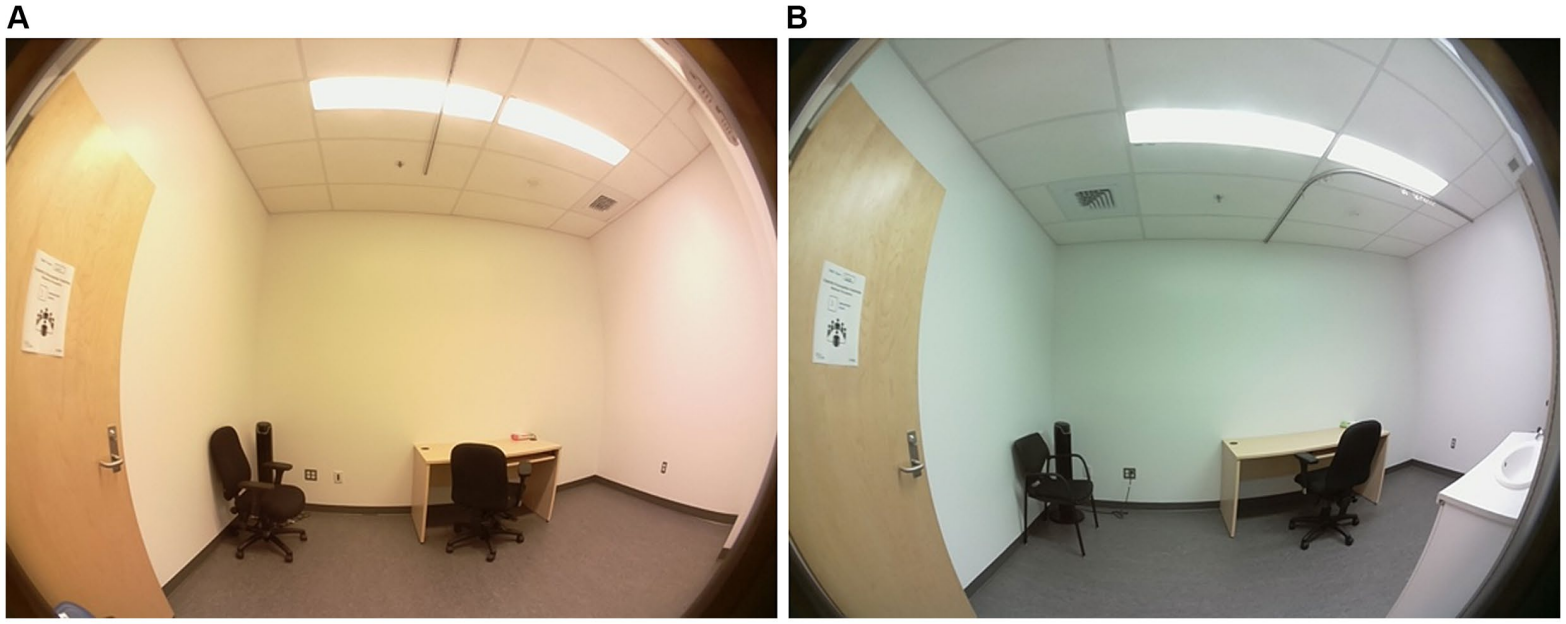
Comparison of the room illuminated by fluorescent lighting (~150 mel-EDI) **(A)** with the room illuminated by LEDs (~260 mel-EDI) **(B)** used in this study. Photo credit Rose Turgeon.

Lighting was provided by two ceiling panels consisting of four 4-foot light tubes. In the control condition room, the current light fixtures were 3,500 K E-Lume^®^ (32) fluorescent tube that yielded to 897 lux (horizontal plane), and 335 lux and 159 mel-EDI (vertical plane). In the experimental room, the conventional fluorescents were replaced with 5,000 K Ecosmart^®^ (33) LED retro-fit tubes that yielded to 837 lux (horizontal plane) and 345 photopic lux and 263 mel-EDI (vertical plane). Therefore, each room provided roughly the same amount of photopic lux, but different mel-EDI levels, with one about 100 mel-EDI below the recommended standard and the other meeting the standards of 250 mel-EDI. Light spectra irradiance were assessed (vertical plane) with the Ocean Insight © spectrometer HR4000 High Resolution (Peabody, MA) (34) and then entered in the CIE toolbox to derive α-opic EDI (lx) illuminances (25). Photopic lux was measured with the ILT5000 research radiometer (International Light technologies, Peabody, MA). Measurements were taken 1.2 m from the ground (4 feet) in the vertical plane for the spectra and photopic lux (35), and in the horizontal plane at 0.76 m from the ground for the photopic measurement only (36). The LED lighting was chosen on the basis that it was commercially available from various hardware stores. Table 1 depicts the α-opic EDI illuminance (lx) for each light condition and Figure 1 represents the α-opic spectra of each room, both obtained with the CIE toolbox (25). For the pre-condition lighting, we used an auxiliary lamp attached to the desk (DAZZNE®, Longhua District, Schenzen) programmed at 3000 K and 50% light power to expose participants to about 25 photopic lux and 10 mel-EDI. The low light intensity used for the neutral condition period was chosen to be sufficient to complete questionnaires without stimulating the melanopsin ipRGCs (17). The advantage of LED light is that it produces a peak in the blue spectrum that closely match the melanopsin cells peak sensitivity and should therefore be very effective in stimulating the melanopsin system while maintaining a whiter looking color. See Figure 2 for the visual impact of both lightings in the different rooms. These lights also consume less energy (20 watts versus 32 watts) and have a longer life span (36,000 h versus 30,000 h for conventional fluorescents). These characteristics make them a wise choice in an energy-efficient context for future applications in the Far North [for instance (37)].

**Table 1:** Photopic lux and α-opic EDI illuminance for each light condition (vertical plane).

Condition	Photopic lux	S-cone-opic	M-cone-opic	L-cone-opic	Rhodopic	Melanopic
Pre-condition	23.1	7.4	18.5	23.5	13.0	**11.1**
CTL (3,500 K)	335.0	129.6	276.5	336.1	189.8	**158.8**
EXP (5,000 K)	344.0	281.0	322.00	341.0	279.2	**263.0**

Bold values means key value of our project.

### 2.5 Variables

The independent variable corresponds to the measurement in mel-EDI of each lighting. The primary dependent variables correspond to the measures of concentration and arousal. These are indeed the two variables of primary interest that we hypothesized would improve significantly more in the experimental lighting when compared to conventional lighting. The secondary variables concern visual acuity, contrast perception and general appreciation of the environment. The vision related variables, while being of interest, were not expected to yield to any difference between the control and experimental lighting. In addition, these tests contributed to fill-up the time during light exposition without increasing the mental load. Information on the appreciation of the room was a relevant variable to collect to verify if the experimental environment with blue-enriched white light receives the same appreciation as the conventional fluorescent lighting room.

### 2.6 Tests and questionnaires

#### 2.6.1 Descriptive questionnaires

Participants completed a set of baseline questionnaires during the pre-condition lighting period. The first session assessed vulnerability to seasonal affective disorder (SAD) using the Seasonal Assessment Questionnaire (38) a screening tool used in research. Those with a score of 11 or higher, perceiving seasonal changes as problematic and feeling worse in January and/or February, were considered to have a vulnerability to SAD (39, 40). Additionally, participants completed the Horne & Östberg Circadian Typology Questionnaire (41), which collects information about the participant’s chronotype. The second session measured sleep quality over the past month with the Pittsburg Sleep Quality Index (42), and excessive daytime sleepiness, also over the past month, with the Epworth Sleepiness Scale (43). These questionnaires were chosen to determine whether they could potentially be used as covariates. In addition, these questionnaires allow us to characterize our sample group.

#### 2.6.2 Subjective alertness

To assess the level of arousal, the Karolinska Sleepiness Scale (KSS) was used. It was designed to assess sleepiness (44) and has been validated to assess alertness and vigilance in sleep deprivation and performance assessment contexts (44–46). It is composed of a Likert scale of 9 items. A score of 1 corresponds to “Extremely alert,” whereas a score of 9 corresponds to “Very sleepy, great effort to keep awake, fighting sleep.”

#### 2.6.3 Concentration

The d2-R test assesses the participants’ concentration. For this test, 658 items are distributed in 14 rows. The items consisted of the letter “d” or the letter “p” with one to four dashes distributed either above or below the letter. The participant had to identify the “d” with 2 dashes (either one above and one below, or 2 below, or 2 above). They had 20 s per row to identify all the correct items, with a total of 14 rows (47–49). This test resulted in two different indicators that were then used in the analysis. On the one hand, the concentration performance (CC) corresponded to the subtraction of the number of errors from the total number of correctly identified items. On the other hand, the measurement of the error rate (E%) corresponded to the proportion of errors in the total number of items analyzed.

#### 2.6.4 Visual performance

Visual performance was assessed using the Freiburg Visual Acuity and Contrast Test (FrACT), which assesses visual acuity as well as contrast perception, using Landolt rings (50, 51). This test was performed on a tablet device with the necessary calibration available on the website. Both visual acuity and contrast perception were tested using a sequence of Landolt rings which varied in orientation and participants were asked to identify this orientation. For visual acuity, the size of the rings was altered and resulted in a LogMAR score (MAR = minimal angle of resolution), which was used in the analysis. Contrast perception was measured by varying the gray shades of the rings, resulting in a LogCS value used in the analysis later.

#### 2.6.5 Visual comfort and preferences assessment

The Office Lighting Survey is a questionnaire created and validated (52) to determine participants’ preferences and visual comfort with workplace lighting. Shamsul et al. (21) used the modified version of the Office Lighting Survey, in order to observe a significant difference in the appreciation of the different lightings as well as in the visual comfort. We used this questionnaire, with the same rating system but translated in French, using DeepL translator^1^ and further validated by two native French speakers (53). This self-administered questionnaire allows participants to indicate to what extent they agree or disagree with different statements about lighting, resulting in two total scores: the preference score and the comfort score.

#### 2.6.6 General lighting appreciation

The second section contained five analogous 100 mm visual scales opposing two adjectives to show the general trend of lighting perception (glaring vs. comfortable, sad vs. cheerful, pleasant vs. unpleasant, sleep-inducing vs. stimulating and dark vs. bright). These analogous scales were based on the questionnaire used by Arsenault et al. (54) when assessing the impact of window glazing.

### 2.7 Study procedure

**Figure 3:**
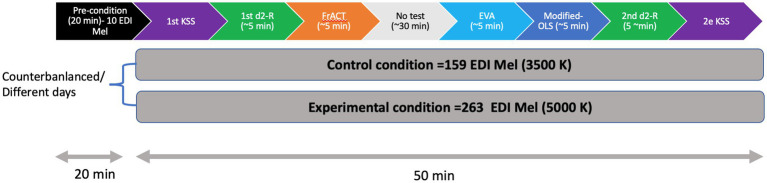
Visual representation of the study procedure.

Each session was conducted in two phases. The first was the 20-min pre-condition period when the descriptive questionnaires were administered. Then, the 50-min light exposure was used to administer the various tests. After the first few minutes of light exposure, the participants took the KSS test once to obtain a baseline value of their sleepiness. Then, they performed the d2-R test to obtain a baseline value of their concentration performance. Afterwards, their visual performance was evaluated with the FrACT test followed by a 20-min period during which they were allowed to read magazines. They were then given the two appreciation questionnaires. The session ended with a second evaluation of the d2-R test and KSS test. Figure 3 provides a visual summary of the protocol.

## 3 Analysis

### 3.1 Questionnaires

Three fixed factors were considered in the analyses: *Group:* Gr 1: participants who began with condition A (n1 = 15); Gr 2: participants who began with condition B (n2 = 15). *Condition:* A: Control lighting with conventional fluorescent light (3,500 K, ~150 mel-EDI); B: Experimental lighting with LED blue-enriched white light (5,000 K, ~260 mel-EDI). *Time:* Pre: at the beginning of the exposure to the condition; Post: at the end of the 50-min exposure to the condition. Table 2 summarizes the items (mean and standard error) found in each of the questionnaires.

**Table 2:** Summary of results (mean and standard error) found in each of the tests and questionnaires.

		Lighting conditions			
		Condition	Mean	SEM	*N*
CC score d2-R	Pre	Control (3,500 K, 159 mel-EDI)	183.90	7.0184	30
		Experimental (5,000 K, 263 mel-EDI)	189.27	7.8943	30
	Post	Control (3,500 K, 159 mel-EDI)	211.03	6.1049	30
		Experimental (5,000 K, 263 mel-EDI)	210.13	6.9662	30
E% Score d2-R	Pre	Control (3,500 K, 159 mel-EDI)	8.3212	1.5644	29
		Experimental (5,000 K, 263 mel-EDI)	7.3639	1.4325	29
	Post	Control (3,500 K, 159 mel-EDI)	7.9747	1.3803	29
		Experimental (5,000 K, 263 mel-EDI)	7.4380	1.2689	29
KSS Score	Pre	Control (3,500 K, 159 mel-EDI)	4.2382	0.349	28
		Experimental (5,000 K, 263 mel-EDI)	4.4882	0.3608	28
	Post	Control (3,500 K, 159 mel-EDI)	3.9167	0.3441	28
		Experimental (5,000 K, 263 mel-EDI)	3.881	0.3561	28
Visual Performance	Visual Acuity	Control (3,500 K, 159 mel-EDI)	−0.1185	0.01924	29
		Experimental (5,000 K, 263 mel-EDI)	−0.1079	0.02048	29
	Contrast Perception	Control (3,500 K, 159 mel-EDI)	1.7767	0.02381	30
		Experimental (5,000 K, 263 mel-EDI)	1.6897	0.03287	30
Modified-OLS	Preference	Control (3,500 K, 159 mel-EDI)	13.9167	0.5081	30
		Experimental (5,000 K, 263 mel-EDI)	11.7833	0.7068	30
	Comfort	Control (3,500 K, 159 mel-EDI)	11.7176	0.6258	30
		Experimental (5,000 K, 263 mel-EDI)	10.8833	0.5102	30
General appreciation *A high score shows a tendency towards the second adjective named in each question*	Glaring vs. Comfortable	Control (3,500 K, 159 mel-EDI)	63.9167	4.9507	30
		Experimental (5,000 K, 263 mel-EDI)	43.0167	5.2434	30
	Sad vs. Cheerful	Control (3,500 K, 159 mel-EDI)	69.8000	3.6932	30
		Experimental (5,000 K, 263 mel-EDI)	56.3000	5.3507	30
	Unpleasant vs. Pleasant	Control (3,500 K, 159 mel-EDI)	70.7500	4.4178	30
		Experimental (5,000 K, 263 mel-EDI)	51.8333	5.9739	30
	Sleep-inducing vs. Stimulant	Control (3,500 K, 159 mel-EDI)	80.9167	2.9166	30
		Experimental (5,000 K, 263 mel-EDI)	86.2667	3.2692	30
	Dark vs. Bright	Control (3,500 K, 159 mel-EDI)	88.4333	2.0627	30
		Experimental (5,000 K, 263 mel-EDI)	95.3167	1.3023	30

### 3.2 Subjective alertness

The factors time and condition were used as repeated measures to establish a possible correlation between the observations made on the same participant. Thus, a 3-factor ANOVA with repeated measures was used. We had to remove two participant’s data since they were considered as extreme values ((i.e., *x* > percentile 75 + 1.5(percentile 75-percentile 25) OR *x* < percentile 25–1.5 (percentile 75-percentile 25)) to allow a normal distribution.

### 3.3 Concentration

The factors condition and time were again used in this 3-factor ANOVA with repeated measures. Both scores, concentration capacity (CC) as well as error rate (E%) were analyzed.

### 3.4 Visual performance

A 2-factor repeated measures ANOVA was used, where the condition factor was considered to establish a potential effect on the same participant. Contrast Sensitivity (in LogCS) and visual acuity (logMAR) were analyzed. One participant’s results were removed from the visual acuity analysis, as they were extreme values, which was calculated in the same way as for the subjective alertness state.

### 3.5 Subjective preference and visual comfort using the OLS-modified scale

The condition factor was used as a repeated measure in the 2-factor ANOVA with repeated measures. The total preference score along with the comfort score were analyzed.

### 3.6 General lighting appreciation using the VAS scale

Again, the condition represented the factor used as a repeated measure in this 2-factor ANOVA with repeated measures. Each of the Visual Analogue Scales (VAS) was analyzed individually.

All the ANOVAs with repeated measures were performed using the MIXED procedure of SAS/STAT software (Version 9.4; SAS Institute Inc., Cary, NC). The graphics in this work were generated using GraphPad Prism 9.3.0 (345) in Viewer mode. GraphPad Prism is a product of GraphPad Software, LLC.

## 4 Results

Table 2 summarizes the items (mean and standard error) found in each of the questionnaires.

### 4.1 Descriptive questionnaires

The average number of days between trials was 3 days with a range of 1–9 days. Sex was not equally distributed with almost twice as many women (*N* = 19) than men (*N* = 11), but there was no difference in age between sexes (*t*-test, *p* = 0.22). The results of the circadian typology show that 63% of the participants belonged to the “neither morning nor evening” category and none of them were in the frankly morning or frankly evening categories. Seasonal affective disorder (SAD) vulnerability was present in 23% of our participants while 33% had excessive daytime sleepiness on the Epworth Sleepiness Scale and 20% had sleep disturbance on the PSQI. None of these findings, however, had a significant impact on the test scores (KSS, d2-R, FrACT, OLS-modified and VAS) in the ANOVA analysis with repeated measures adjusted for co-variables.

### 4.2 KSS

**Figure 4:**
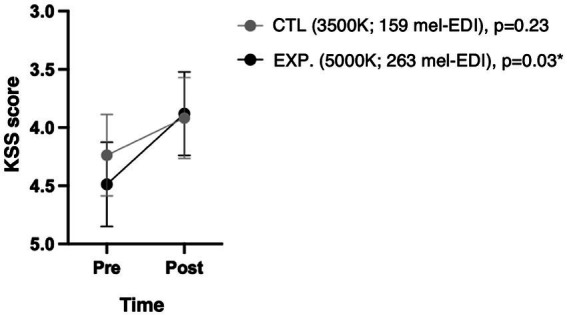
Subjective sleepiness scale (KSS) according to condition and time. Because the KSS score is such that a state of alertness corresponds to a lower score, the y-axis has been reversed to be visually intuitive to readers. The *p*-values shown are the changes over time under each condition.

Both lighting conditions yielded to some improvement in alertness over the 50-min light exposure, but reach significance only in the experimental light condition (*p* = 0.03, Cohen’s d = 0.42) (Figure 4).

### 4.3 Concentration capacity (CC score)

**Figure 5:**
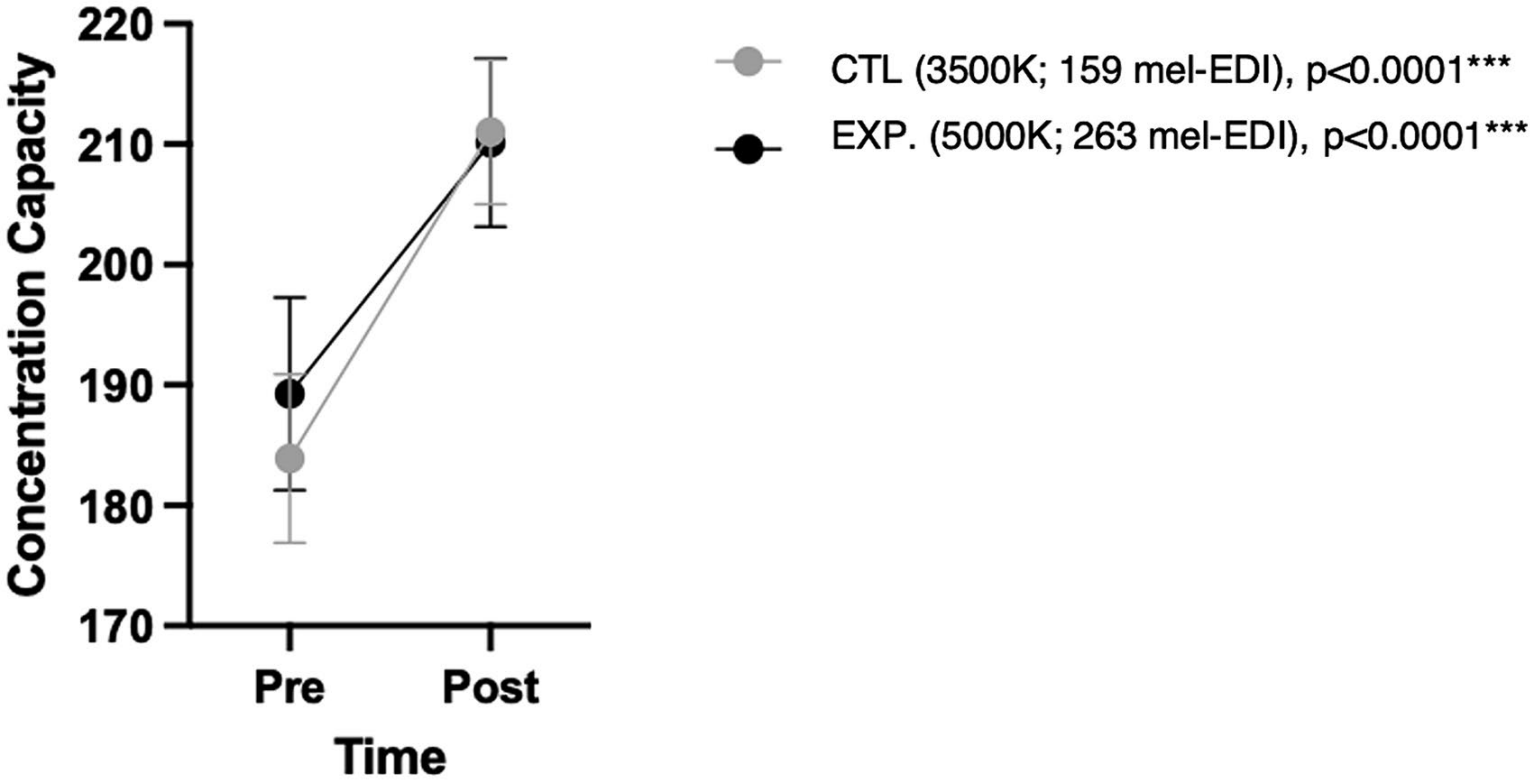
Concentration capacity in d2-R test according to condition and time. *p*-values shown correspond to changes over time under each condition.

Subjects significantly improved their concentration performance at the end of the 50-min exposure in both conditions (*p* < 0.0001) with no significant difference between conditions (*p* = 0.79), as shown in Figure 5.

### 4.4 Error rate (E%)

One participant’s results had to be removed because all data were outliers. No factor demonstrated a statistically significant change over the 50-min exposure. Thus, participants did not improve by time nor condition (*p* = 0.40 and *p* = 0.97, respectively).

### 4.5 Visual acuity

No statistically significant difference was shown between the two conditions during the 50-min light exposure (*p* = 0.61).

### 4.6 Contrast sensitivity

When exposed to the experimental, blue-enhanced light (5,000 K, ~260 mel-EDI), participants performed significantly worse on the contrast perception test when compared to their performance in the control lighting (3,500 K, ~150 mel-EDI) (*p* = 0.01, Cohen’s d = 0.50).

### 4.7 Subjective preference

**Figure 6:**
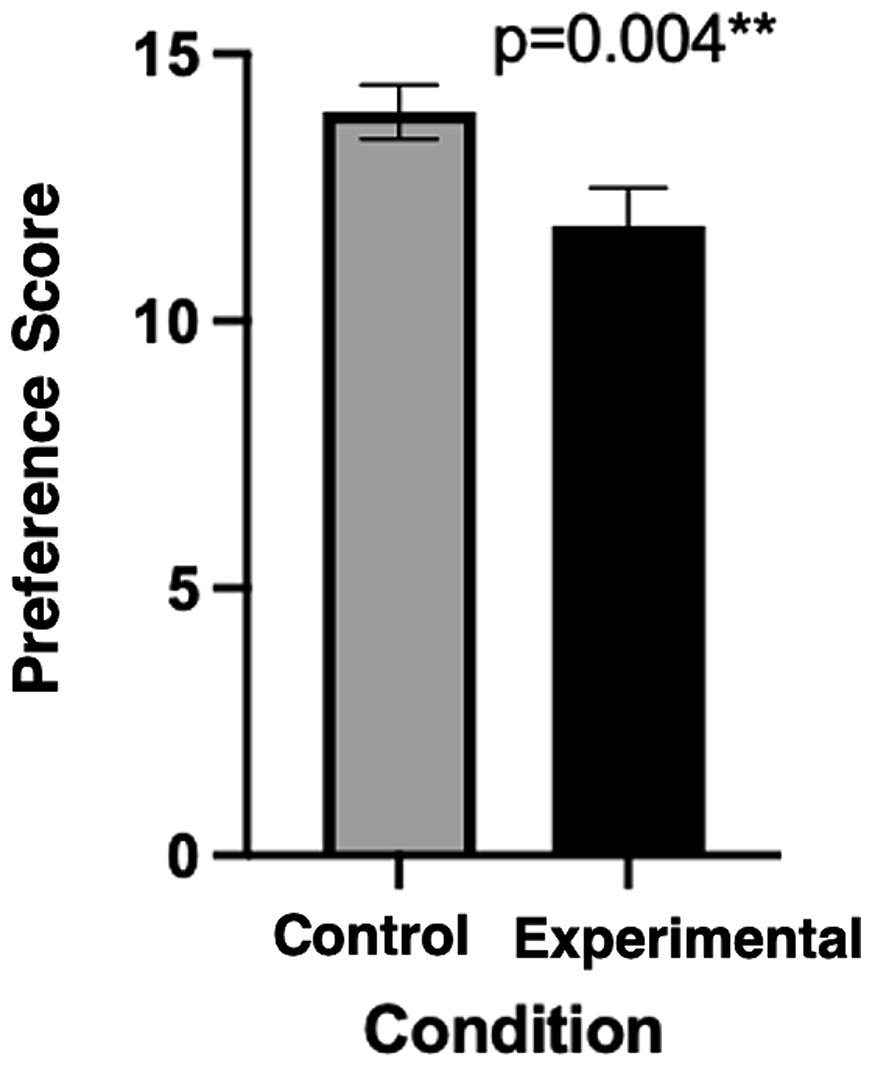
Preference score using the modified-OLS between both conditions.

**Figure 7:**
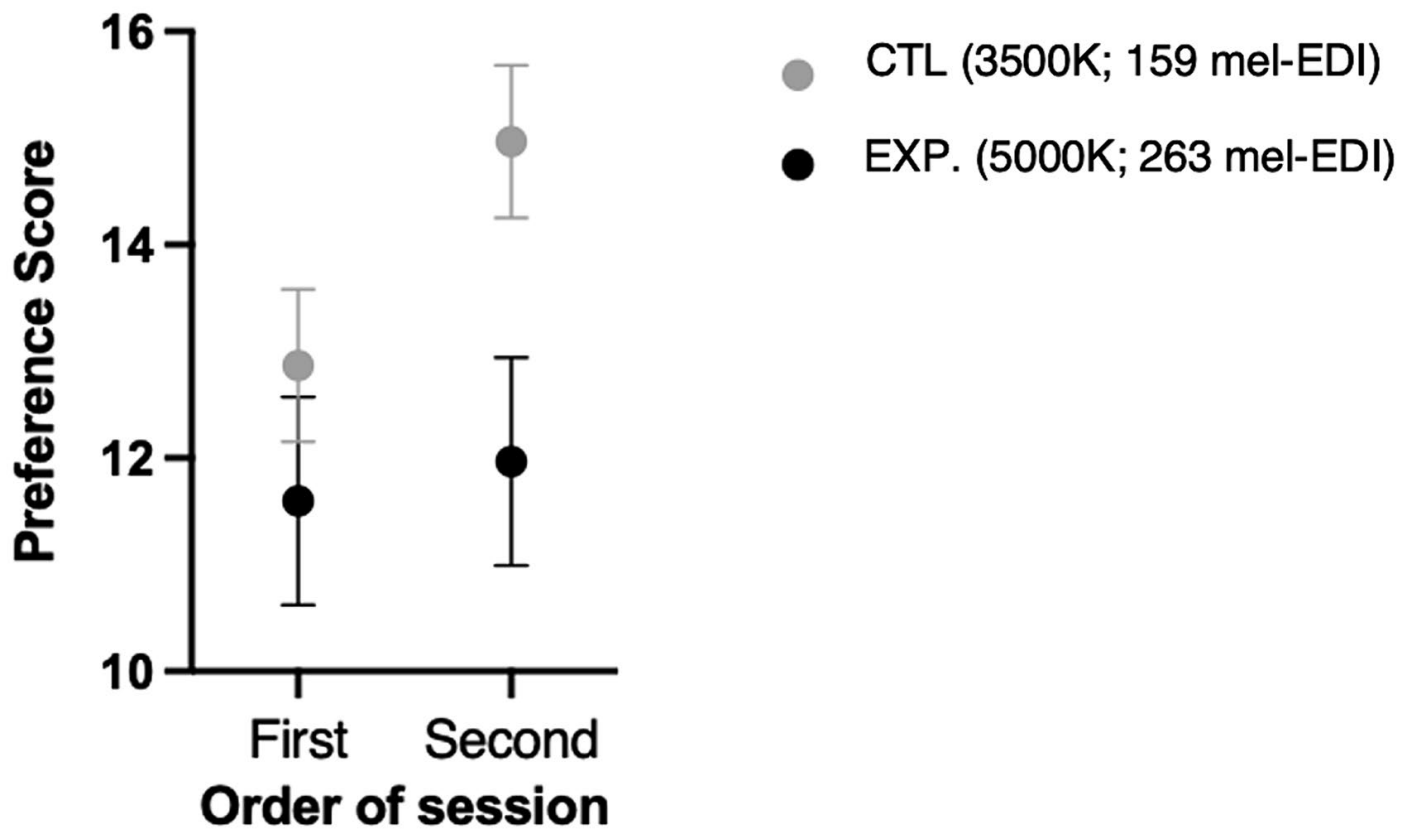
Preference score using the modified-OLS related to order of passage.

Participants significantly preferred the control room (3,500 K, ~150 mel-EDI) compared to the experimental lighting (5,000 K, ~ 260 mel-EDI) (*p* = 0.004, Cohen’s d = 0.56), as shown in Figure 6. However, a positive interaction was noted regarding the order of exposure. Thus, participants who started with the experimental condition tended to increase the preference score when they were asked to evaluate the control room to which they were exposed secondly (*p* = 0.07). Figure 7 illustrates this interaction.

### 4.8 Visual comfort

The last question of the questionnaire was not considered since it involved difficulty reading items on the screen. Since the only test required on-screen was visual performance, this question was inapplicable with the setup of our study. Overall, there was no significant difference between the two conditions for visual comfort (*p* = 0.17).

### 4.9 General lighting appreciation

The control fluorescent-lit room (3,500 K, ~150 mel-EDI) was found to be significantly more comfortable (*p* = 0.002, Cohen’s d = 0.62), more cheerful (*p* = 0.02, Cohen’s d = 0.46) as well as more pleasant (*p* = 0.005, Cohen’s d = 0.55). The experimental LED room (5,000 K, ~260 mel-EDI) was perceived as brighter (*p* = 0.004, Cohen’s d = 0.58) and tended to be perceived as more stimulating (*p* = 0.10). In terms of the degree of pleasantness as well as the degree of cheerfulness rated by participants, a positive interaction was noted in relation to the order in which they were exposed. Similarly, to the lighting preference noted in the modified OLS questionnaire, participants who started with the experimental condition tended to rate a higher level of pleasantness for the control room on their second encounter (*p* = 0.03). The same goes for the level of cheerfulness (*p* = 0.01).

## 5 Discussion

The present study shows that a short exposure to ~260 mel-EDI (5,000 K) results in a significant improvement in the subjective state of alertness, with a tendency to perceive it as more stimulating, as expected. On the other hand, ~150 mel-EDI (3,500 K) was found to be more comfortable, pleasant, and cheerful. However, concentration was not further improved by exposure to light meeting mel-EDI standards when compared to conventional lighting.

The finding on subjective alertness using the KSS is consistent with prior research indicating heightened alertness after a 60-min exposure to 6,000 K lighting (21, 22). However, the effect size observed in our study, falling within the moderate range, suggests that while statistically significant, the practical impact of this reduction in sleepiness may be modest. This could partly account for why concentration (CC) did not show further improvement in the blue-enriched light condition. It’s plausible that the difference in mel-EDI was not significant enough, or that the concentration enhancement wasn’t solely due to lighting conditions but could be influenced by a learning effect or a deeper task understanding. To address this, implementing a training task during the pre-condition period or on the previous day could have minimized these factors in subsequent performance. Our study contrasts with another study where concentration task improvement was more pronounced after short exposure to blue-enriched lighting compared to traditional lighting (20). Notably, their study design differed, lacking a cross-over design and having varied group sizes and ages. Moreover, details on the photopic lux level for each lighting condition were unavailable, unlike our study where photopic lux levels were kept similar. This difference in photopic lux levels could also contribute to variations in concentration task outcomes (20).

The visual analog scales provided subjective insights into the two illuminations. Specifically, the experimental LED lighting (5,000 K, ~260 mel-EDI) was perceived as brighter and more stimulating, aligning with the subjective feeling of cognitive stimulation also captured by the KSS. However, the modified OLS questionnaire revealed a preference for the control lighting (3,500 K, ~150 mel-EDI) over the experimental lighting (5,000 K, ~260 mel-EDI). This preference echoes findings from previous studies (21) where higher CCT lightings were generally disliked. Similarly, studies have shown a preference for 4,000 K lighting over 5,000 K and 3,000 K, although these comparisons were within fluorescent lamps (55). We aimed for the LED light (5,000 K) to approximate the usual color temperature (3,500 K-4000 K) to ensure equivalent appreciation for both lights. Our rationale for using LED was based on its spectrum, which balances the short-wave peak stimulating melanopsin with longer wave peaks (red-green). This concept aimed for a comprehensive approach where sufficient mel-EDI stimulation could be achieved while maintaining illumination equivalent to usual lighting in terms of photopic lux. Moreover, existing evidence indicates that LED lighting (trial of 2 h) is generally preferred and provides greater comfort compared to both fluorescent lighting with the same CCT (6,500 K) and conventional fluorescent lighting with a CCT of 3,500 K (28).

Moreover, blue-enhanced lighting provides was also perceived as more sad, unpleasant, and glaring. These adjectives therefore match the lowest preference score for experimental lighting found in the modified OLS questionnaire. The fact that the group that was first exposed to the experimental (5,000 K, ~260 mel-EDI) lighting showed a greater representation of the “happy” and “pleasant” aspects of the control (3,500 K, ~150 mel-EDI) lighting when compared to the group that started with the conventional lighting clearly indicates that a comparison (even not on the same day) allows for a more accurate overall assessment. This finding also correlates with the significantly increased preference of conventional lighting (in the modified-OLS questionnaire) in the group exposed to it in the second session compared to the group exposed to it in the first session.

Visual comfort was however similar in both conditions, which is consistent with similar studies (21, 56). Moreover, no difference in visual acuity was demonstrated, which is consistent with our initial hypothesis. Since photopic lux represents the light intensity stimulating the receptors responsible for vision, it stands to reason that our two conditions provided proper stimulation of the visual system (57). It should also be noted that the WELL standards recommend a minimum horizontal illuminance of 300 photopic lux (horizontal plane) to maintain adequate visual acuity (36). Given that the horizontal illuminance was similar between conditions (i.e., 897 lx for 3,500 K and 837 lx for 5,000 K), it is not surprising to observe no significant difference in the visual acuity task between both conditions (36). However, our ability to see depends not only on visual acuity but also on the ability to perceive contrast. Therefore, the poorer contrast perception in the blue-enhanced white light condition is contrary to our hypothesis. This finding may be related to the fact that participants found this lighting subjectively too bright due to the increased presence of blue wavelengths. Since the assessment of contrast perception was always performed after the assessment of visual acuity, it is possible that some ocular discomfort due to the sensation of glare may have affected contrast perception (58). Recent studies suggest that a reduction in contrast sensitivity of 0.3 logCS units would be consider to have a clinically significant impact on the contrast perception task (59). Thus, it appears that our mean reduction of 0.087 logCS, although statistically significant with moderate effect size, does not reach clinical significance. It should be noted that the exposure time was quite short, so we cannot speculate what would happen after a full day of working.

## 6 Limitations

### 6.1 Factors contributing to general appreciation

Although our short exposure study seems to favor the use of conventional fluorescent light, it would be interesting to evaluate the general appreciation of an environment lit by a blue-enriched LED light over a longer period as done by Viola et al. (19). In this way, participants would have more time to accustom themselves to the lighting and potentially improving appreciation for it, as was shown in their study. Moreover, a more recent study comparing a 130-min exposure to 5,700 K and 2,700 K demonstrated improved arousal and attention performance under 5,700 K compared to 2,700 K, though without a significant impact on visual comfort. In fact, participants reported fewer eye-related symptoms under 5,700 K lighting (56). In addition, it is possible that the high reflectance of the white walls (about 80%) had an impact on the overall appreciation of the lighting. Indeed, it seems that white walls allow a better appreciation of warm colored lighting (lower CCT), while blue painted walls allow a better appreciation of cold colored lighting (higher CCT), which would explain why participants preferred the control (3,500 K, ~150 mel-EDI) lighting when compared to the experimental (5,000 K, ~260 mel-EDI) lighting (60). Perhaps a similar study with blue walls for cooler-looking lighting would balance the preferences between the two rooms, although more light would be needed to reach the 250 lux mel-EDI threshold since colored walls do not reflect as much light as white walls.

### 6.2 Controlled environment

Another consideration is that there were no windows in the rooms studied, which allowed for total control of melanopic and photopic intensities. On the other hand, these conditions do not represent the full reality of contemporary architecture, where biophilia associated with daylighting is an integral part of building design (61). It may therefore be that the preference for blue-enriched lighting in the Viola et al. (19) study is a combination of chronic exposure time associated with more biophilic components such as natural light.

### 6.3 Population specificity

An important consideration to bear in mind is that our study is currently centered on the Quebec population. It is conceivable that Far North populations might not respond in the same manner due to distinct customs and lifestyle habits that may result in more outdoor activities. Additionally, environmental factors need to be considered, such as the unique conditions in Quebec City where cloud cover might scatter sunlight differently compared to other regions. These cultural and environmental variables could potentially influence how individuals from Far North regions and Quebec City respond to the lighting conditions studied in our research. Further investigation and studies encompassing diverse populations and geographical locations may provide a more comprehensive understanding of the impacts of lighting on different communities. Furthermore, the study population comprised predominantly of university students, limiting its applicability to the working population with potentially higher age demographics. A more precise representation of the working population’s age would be desirable for future replications.

### 6.4 Room ergonomics

Although the rooms used do not meet the ergonomic and architectural recommendations for offices, they allowed this study to be conducted in a controlled environment. In fact, we were able to recreate an environment with a positioning that allowed participants to be exposed to the 250 mela-EDI standard under one light, while the other light was short of 100 lux below the mel-EDI exposure standard. For example, the desk was positioned so that participants had their backs to the light source and received their exposure primarily through the wall (by reflection), whereas in actual practice this exposure should be more directly in the field of view with a desk positioned under the light fixture. This positioning could also give the impression that the work surface is not sufficiently illuminated, thus reversing the illuminance ratio for performing a task. In addition, this study did not account for the effect of partial light masking of participants when seated at a desk. In addition, because participants did not work on screens except for the very brief FrACT test, the reflection of light off a screen (which would also contribute to enhanced blue light exposure) is not accounted for and should be considered in future studies involving computer work.

### 6.5 Absence of baseline measures

This study did not include baseline measures. As indicated in a study by Smolders and De Kort (62), participants may exhibit variations in baseline responses on different days. The absence of baseline measurements in our study leaves us unable to account for or assess the potential impact of this day-to-day variability on participants’ responses to different lighting conditions.

## 7 Strengths

Our study allowed us to compare results within subjects, so we were able to specifically evaluate how light affected each test and limit individual performance biases. In addition, conducting the assessments at the same time of day among participants limits the effects of circadian variations on alertness (30). To limit the effects of changing weather on cognitive status, patients also had a leveling period before their assessment. Likewise, it is possible to assess the stimulating effect of light for the specific period targeted by this study, since all participants took part in the study during the same season, i.e., late fall. The fact that the assessments were all made in the same season also limits the bias that may result from an increase in photoperiod availability that differs from season to season. Another strength is that both of our lights had the same photopic lux level, limiting the potential effects of variation in this measure on cognition.

## 8 Conclusion

This study suggests that a brief exposure (50 min) to light following the recommended standards for mel-EDI (17) leads to a significant improvement in subjective alertness. However, it does not lead to a further improvement in concentration or performance accuracy over conventional lighting. Moreover, participants reported dissatisfaction with the 5,000 K LED lighting, finding it overly blue-toned. This study serves as an initial exploration before a more extensive investigation, allowing participants time to adapt to the blue hue of the lighting, potentially leading to greater appreciation while confirming the positive findings of our short-term exposure.

## Data availability statement

The original contributions presented in the study are included in the article/supplementary material, further inquiries can be directed to the corresponding author.

## Ethics statement

The studies involving humans were approved by CIUSSS de la Capitale-Nationale Neuroscience and Mental Health Research Ethics Committee. The studies were conducted in accordance with the local legislation and institutional requirements. The participants provided their written informed consent to participate in this study.

## Author contributions

VG: Conceptualization, Data curation, Formal analysis, Investigation, Methodology, Project administration, Writing – original draft, Writing – review & editing. RT: Data curation, Methodology, Writing – review & editing. VJ: Formal analysis, Writing – review & editing. CD: Conceptualization, Funding acquisition, Resources, Supervision, Validation, Writing – review & editing. MH: Formal analysis, Writing – review & editing, Conceptualization, Data curation, Funding acquisition, Investigation, Methodology, Project administration, Resources, Software, Supervision, Validation, Visualization.

## Footnotes

^1^ www.deepl.com/translator
